# 481. SARS-CoV-2 Infection in Hospitalized Children: An Elevated Body Mass Index is a Marker of Increased Risk of Acute Respiratory Failure

**DOI:** 10.1093/ofid/ofab466.680

**Published:** 2021-12-04

**Authors:** Catherine Foster, Shelley Kumar, Elizabeth Tocco, Galit Holzmann-Pazgal, Judith R Campbell, Lucila Marquez, Ankhi Dutta

**Affiliations:** 1 Baylor College of Medicine, Houston, Texas; 2 Texas Children’s Hospital, Houston, Texas

## Abstract

**Background:**

Several risk factors are known to increase the severity of coronavirus disease 2019 (COVID-19) illness in adults, including age and obesity. Specific comorbidities affecting COVID-19 outcomes in children are less well defined.

**Methods:**

We performed a retrospective cohort study of overweight and obese (OW) children compared to underweight and normal weight (NW) children with severe acute respiratory syndrome coronavirus-2 (SARS-CoV-2) infection. Children between 2 and 18 years of age who were admitted to Texas Children’s Hospital from April through December of 2020 with a positive SARS-CoV-2 polymerase chain reaction test were included. Asymptomatic patients undergoing surveillance testing for SARS-CoV-2 were excluded. Body mass index (BMI) was calculated using the Centers for Disease Control definition. Demographic and clinical information was obtained from the electronic medical record. Statistical analyses were performed using SAS 9.0.

**Results:**

We identified 145 total children who met inclusion criteria. Fifty-five (38%) children were NW and 90 (62%) children were OW. Demographics and characteristics are shown (Figure 1). Underlying asthma or chronic lung disease was present in 13 (24%) vs 31 (34%) in the NW and OW groups respectively (*P*=0.17). OW children were more likely to have pneumonia than NW children [relative risk1.6 (CI 1.40-2.45)]. An elevated BMI was also associated with an increased risk of requiring oxygen [relative risk 1.4 (CI 1.03-1.96)]. The median length of hospitalization was 4 days for NW versus 5 days for OW children (*P*=0.6). Admission to the Intensive Care Unit (ICU) was similar between the groups (*P*=0.7). There was no significant difference in treatments administered to children in the two groups, although there was a trend towards increased steroid (29 (53%) vs 59 (67%), *P*=0.13) and remdesivir (12 (22%) vs 30 (33%), *P*=0.14) use in the OW children. Four children in each group died.

Characteristics of Hospitalized Children with SARS-CoV-2 Infection by Weight Category

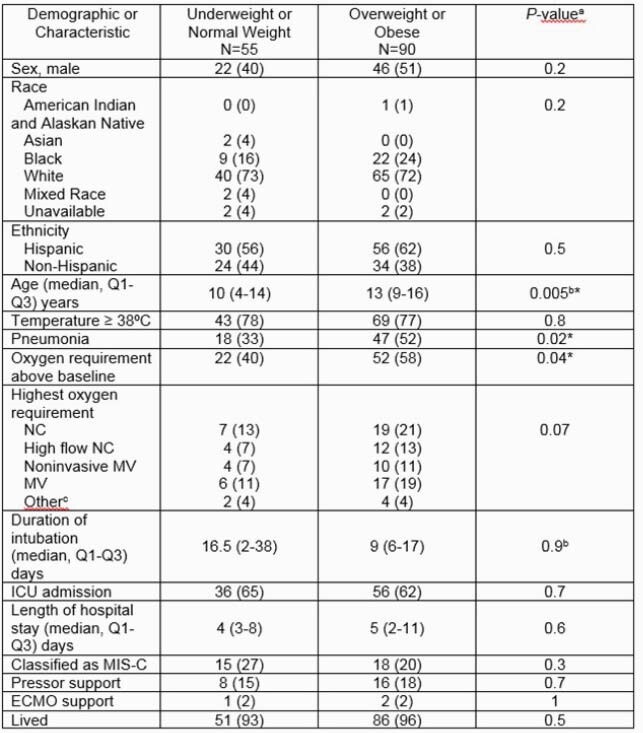

Abbreviations: ECMO, extracorporeal membrane oxygenation; ICU, intensive care unit; MIS-C, multisystem inflammatory syndrome in children; MV, mechanical ventilation; NC, nasal cannula *Denotes statistically significant P-value a. Calculated using chi-square or fisher exact unless otherwise noted. a. Calculated using chi-square or fisher exact unless otherwise noted. A P-value <0.05 was considered significant. b. Calculated using Wilcoxon rank sum test. c. Includes patients with home noninvasive MV (2) or tracheostomy and home MV(4).

**Conclusion:**

For children admitted with symptomatic COVID-19, being overweight or obese was significantly associated with having pneumonia and with requiring oxygen. A difference in ICU admission, length of hospitalization, and mortality was not observed. Obesity prevention along with vaccination efforts may prevent COVID-19 related morbidity in this group.

**Disclosures:**

**All Authors**: No reported disclosures

